# Risk factors of metabolic syndrome among adult Sudanese sickle cell anemia patients

**DOI:** 10.1186/s12878-018-0110-7

**Published:** 2018-12-27

**Authors:** Awab Omer Babiker, Lamis AbdelGadir Kaddam

**Affiliations:** Department of Physiology, Faculty of Medicine, Alneelain University, P.O. Box: 11121, 12702 Khartoum, Sudan

**Keywords:** Metabolic syndrome, Hyperlipidaemia sickle cell anemia, Steady state

## Abstract

**Background:**

Sickle cell disease is a hereditary disorder characterized by haematological anaemia. Several studies assumed that adult sickle patients might develop metabolic syndrome features as hyperglycaemia, hypertension and dyslipidaemia. The aim of this study was to evaluate the metabolic syndrome risk factors among adult Sudanese with sickle cell anemia in the steady state.

**Methods:**

A prospective cross sectional study design was conducted among thirty adult patients with sickle cell anemia Hb SS (mean age 23 ± 6.1 years) and thirty healthy individuals matched for age and gender. Waist and hip circumferences were measured by simple tape. Venous blood sample were analysed to detect blood glucose level, uric acid, total cholesterol, triglycerides, low and high-density lipoprotein after 8 h overnight fasting by spectrophotometer. Blood pressure was measured by sphygmomanometer. National Cholesterol Education Program-Adult Treatment Panel III was utilised to define metabolic syndrome. Statistical analysis was performed SPSS software version 23. Continuous data were expressed using mean ± SD. *P*-value of < 0.05 (two-tailed) was used to establish statistical significance. Unpaired independent T- test was used.

**Results:**

No significant difference in mean systolic blood pressure in patients group compared to control (*P* value = 0.3). Mean value of diastolic blood pressure was significantly low in patients group compared to control (65.4 ± 10. 4 VS72.33 ± 8.27 mmHg, *P* value< 0.001). Fasting triglycerides level was comparable between patients group and control (P value = 0.56). While high-density lipoprotein was significantly lower in sicklers compared to control (30.2 ± 8.2 mg/dL vs 44.71 ± 1.85 mg/dL, *P* value< 0.001). Fasting blood glucose was significantly low in sickle compared to control (92.6 ± 13 mg/dL vs 106.83 ± 25.11 mg/dL P value< 0.001). Uric acid level was not statistically differed in patients group compared to control (*p* value = 0.5).

**Conclusion:**

There was significant decrease in fasting High-density lipoprotein, diastolic blood pressure, mean arterial pressure and fasting blood glucose among SCA patients compared to control. There was no significant difference in waist circumference, systolic blood pressure, fasting triglycerides and uric acid levels between patients and control groups.

## Background

Sickle cell anemia (SCA) is a genetic haematological disorder caused by mutations in the sixth amino acid to be substituted from glutamic acid to valine and characterized mainly by vaso-occlusion and anaemia [1]. Nowadays several researches investigate metabolic syndrome (MetS) development among SCD patients [2]. Little is known, however, about the prevalence of metabolic syndrome determinants among adults with SCD [2]. Metabolic syndrome is a cluster of conditions that are associated with a two-fold increase in cardiovascular disease outcomes and a 1.5-fold increase in mortality [2, 3]. To diagnose the metabolic syndrome, patients must achieve three out of five factors: increased waist circumference, triglyceride levels, blood pressure and fasting blood glucose levels, and decreased HDL levels [2, 3].

Waist circumference was found to be lower in patients with SCD, although waist to hip ratio is greater than their matched control; which is most probably due to recurrent avascular necrosis of head of the femur that causes a decrease in hip circumference [4]. SCD is associated with hypocholestrolemia when compared to control group [1]. They found low level of low density lipoprotein cholesterol (LDL), low level of high density lipoprotein (HDL) and high triglycerides level [1]. Two latter parameters by their powerful atherogenic potential can possibly carry an early cardiovascular risk [1]. Recent study showed that patients with (SCD) may develop insulin resistance [5]. Insulin resistance might be due to high Reactive Oxygen Species (ROS) and decrease antioxidants or increase ferritin level [5]. Sickle cell disease has a great effect on blood pressure [6]. Patients have decreased diastolic blood pressure and increased pulse pressure, and the systolic blood pressure might be higher compared to control [6]. The significantly lower diastolic blood pressure and increased pulse pressure noted in patients could be as a result of the hyperdynamic circulation from chronic anaemia [6]. Although these differences were not observed when the effect of anaemia was eliminated by comparing patients and controls with mild anaemia [6]. Also sickle cell disease may be associated with hyperurecimia which could be due to increased catabolism of nucleic acid [7].

Sickle cell disease patients develop high reactive oxygen species due to higher autoxidation of HbS generating superoxide anion radicals and hence hydrogen peroxide, which it has great effects on serum blood glucose and lipid profile [5]. These will increase the rates of metabolic syndrome with its complications.

Sickle cell disease nowadays categorized as syndrome rather than a disease. It affects many organs and can be consider as a life threatening condition. According to National Cholesterol Education Program, we measured waist circumference, fasting blood glucose, fasting lipid profile and blood pressure as characteristics of metabolic syndrome.

## Subjects and methods

### Study design

This is a prospective observational descriptive cross sectional study design. It was conducted in adult haematological clinic Military hospital, Omdurman, Khartoum state.

Adult Sudanese with sickle cell anemia in the steady state attended haematology clinic during the period from August to November 2017. Total number of patients during that period was thirty. Thirty Medical students of Alneelain University participated as a control group selected matching in physical characteristics to patients group. The study included all adult SCA patient in steady state-Patients free of crises in the last 12 weeks- attending to haematological clinic in military hospital during study period. All patients were homozygous for SCD (SS) as documented by Hemoglobin electrophoreses. Any SCA patients with congenital or acquired heart disease, pregnant women, very severe anemia (hematocrit < 18%), known case of renal diseases or on medications other than folic acid and hydroxyurea were excluded from the study.

### Methods of data collection

Interviewed questionnaire was used, which was subdivided into: Background of patients (age, gender, marital status, education level, tribe and residency) and past medical history of sickle cell anemia.

#### Anthropometric measures

Weight was measured by electronic scale in kilograms. Participants were standing, heavy clothes were taken off and absolute empty pockets. The weight was approximated by decreasing 0.5 kg from participant’s readings, clothes were considered. Height, waist and hip circumferences were measured by simple tape. Waist circumference was measured three times at the level of umbilicus, between lower margin of the lowest rib and superior surface of iliac crest, the mean value was considered as a reference value. The tape was placed three times around the widest area at hip region to measure hip circumference. Body mass index was calculated using this equation: BMI = weight (kg) / Height2 (m). Grading of BMI was done according to WHO grading (normal values: 18.5–24.9 kg/m2, below 18.5 are underweight, individuals with BMI ranging from 25 to 29.9 are overweight BMI, above 30 are labeled obese and those with BMI more than 35 are morbid obese).

#### Blood pressure

Was measured by mercury sphygmomanometer with suitable cuff; covering two thirds of upper arm, applied 2.5 cm above the cubital fossa, brachial artery was localized well, patients were sitting in comfortable temperature.

#### Blood samples

Venous blood sample of 6 ml after 8 h overnight fasting was drawn, distributed in two tubes one of lithium heparin for clinical chemistry (Lipid profile, uric acid and FBG) done by spectrophotometer, and other of EDTA for cell counting. NCEP ATPIII definition was used to diagnose metabolic syndrome.

Definition of metabolic syndrome according to NCEP – ATP III, if three factors of the followings were achieved [3]:

**Table:** 

Risk factor	Defining level
Waist circumference	- Men > 102 cm - Women > 89 cm
Blood pressure	- Systolic ≥130 mmHg - Diastolic ≥85 mmHg
HDL	- < 40 mg/dL
Triglycerides	- > 150 mg/dL
Fasting blood glucose	- > 110 mg/dL

Statistical analysis was performed using SPSS software version 23. Continuous data were expressed using mean ± SD. *P*-value of < 0.05 (two-tailed) was used to establish statistical significance. Unpaired independent T- test was used.

## Results

This study was done on thirty patients with Hb SS and thirty medical students Hb AA to study the determinants of metabolic syndrome. Table 1 displays the background of both sickle cell anemia patients and controls. Age showed no significant difference between patients and control group (23.06 ± 6.06 vs 20.73 ± 1.81 respectively, *p* value = 0.05). Gender showed no significant difference between two groups.

**Table 1:** Background characteristics of Participants

Characteristics	subjects			*P* value
	Sickle patients (N = 30) (mean ± SD)	Control (*N* = 30) (mean ± SD)	Total (*N* = 60)	
AGE IN YEARS	23.06 ± 6.06	20.73 ± 1.82	22 ± 4.59	0.05
GENDER: MALES	13(43.3%)	11 (36.7%)	24 (40%)	0.23

In Table 2 Body Mass Index (BMI) showed no statistical difference between patients and control group (20.8 ± 3.1 vs 22.10 ± 4.56 respectively, *p* value = 0.2). Waist circumference showed non-significant difference between patients group compared to control group (p value = 0.15). Hip circumference was significantly lower in patients group compared to control group (*p* < 0.001). Waist-hip ratio was significantly higher among patients group compared to control (*p* = 0.001).

**Table 2:** Anthropometric Measurements of Participants

Characteristics	subjects			*P* value
	Sickle patients (N = 30) (mean ± SD)	Control (N = 30) (mean ± SD)	Total (N = 60) (mean ± SD)	
Body Mass Index	20.8 ± 3.1	22.10 ± 4.56	21.5 ± 3.67	0.20
Waist circumference (cm)	69.77 ± 7.16	73.30 ± 11.07	71.53 ± 9.41	0.15
Hip circumference (cm)	79.8 ± 5.13	95.6 ± 12.29	87.2 ± 11.90	0.00^a^
Waist - hip ratio	0.86 ± 0.49	0.78 ± 0.11	0.78 ± 0.1	0.00^a^

^a^Statistically significant

Table 3 displays that the SBP with no significant difference between patients and control groups (*P* value = 0.31). DBP was significantly low in SCD (65.4 ± 10. 4 mmHg) compared to control (72.33 ± 8.27), *p* value> 0.001. PP was not differ in two groups (40.70 ± 1.13 mmHg vs 37.23 ± 9.87 mmHg respectively, p value = 0.23). MAP was lower in patients compared to control (79 ± 1.16 mmHg vs 84.30 ± 7.57 mmHg, respectively. *P* = 0.05).

**Table 3:** Blood Pressure among Participants

Variable	Sickle patients (N = 30) (mean ± SD)	Control (N = 30) (mean ± SD)	Total (N = 60) (mean ± SD)	*P* value
SBP	106.1 ± 16.4	109.6 ± 9.09	107.9 ± 13.27	0.31
DBP	65.4 ± 10. 4	72.33 ± 8.27	68.88 ± 9.96	0.00^a^
PP	40.7 ± 1.13	37.2 ± 9.8	39.1 ± 1.1	0.23
MAP	79 ± 1.16	84.3 ± 7.57	81.47 ± 1.02	0.05

^a^Statistically significant

**Fig. 1:**
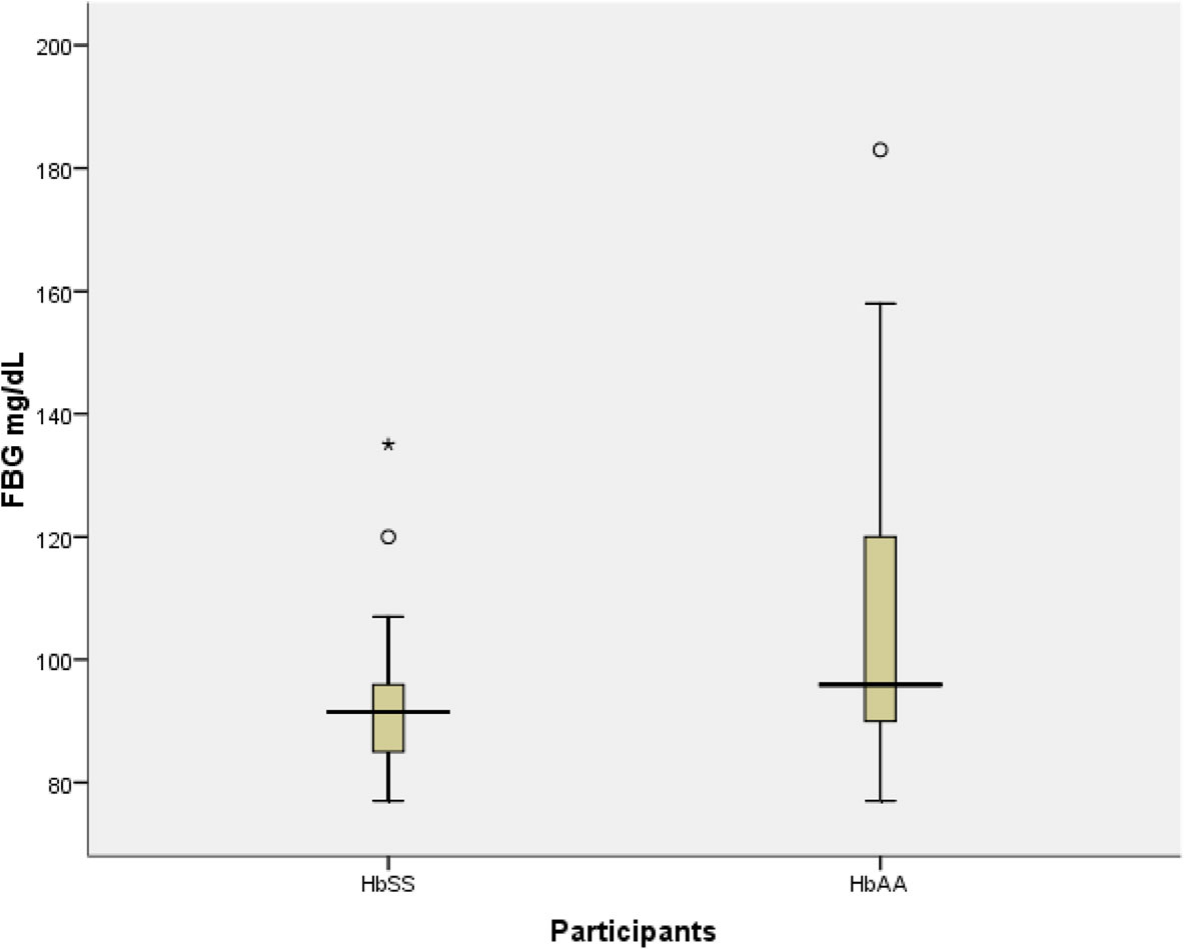
Fasting Blood Glucose in Sickle Cell Disease Patients Compared to Control

**Fig. 2:**
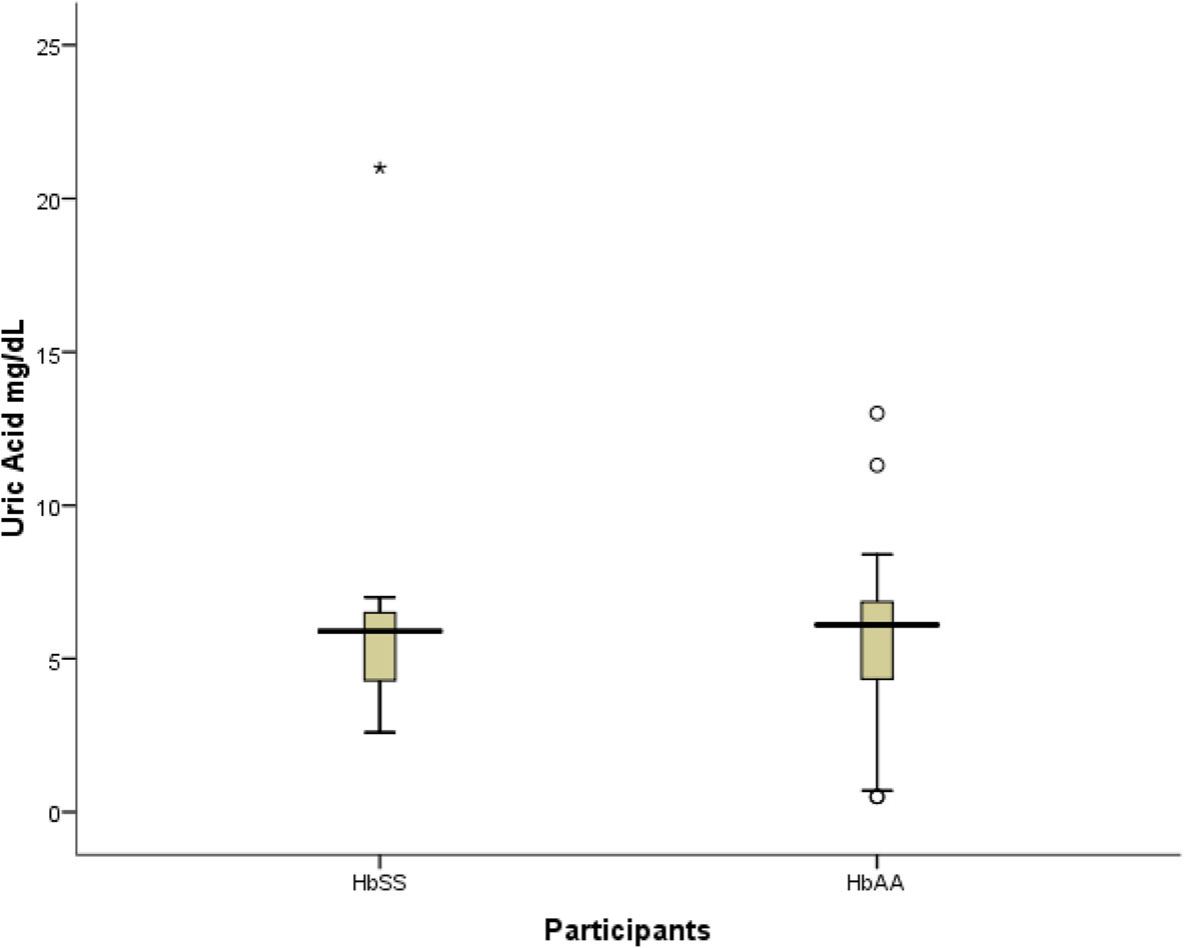
Uric Acid Level in Sickle Cell Disease Patients Compared to Control

Table 4 represents the levels of fasting lipid profile in patients and their matched control group. The fasting cholesterol level was not statistically high in patients compared to control group (*p* value = 0.17). There was no significant difference in F.TG between patients and control group (p value = 0.56). Both HDL and LDL level were significantly lower in patients group compared to control group (p value =0.00,p value = 0.00) respectively. Fig. 1 demonstrates fasting blood glucose level is lower in patients compared to contro. (*P* value = 0.01). Fig. 2 shows no significant difference in serum uric acid among patients and control groups (6.0 ± 3.6 mg/dl vs 5.52 ± 2.93 mg/dl respectively, *p* value = 0.56).

**Table 4:** Fasting Lipid Profile in Participants

Biomarker	Sickle patients (N = 30) (mean ± SD)	Control (N = 30) (mean ± SD)	Total (N = 60)	*P* value
F. Cholesterol	112.3 ± 43.89	98.34 ± 29.96	104.56 ± 37.10	0.17
F. TG	82.8 ± 3.96	91.37 ± 6.23	87.53 ± 5.29	0.56
F.HDL	30.2 ± 8.15	44.71 ± 1.85	37.49 ± 1.59	0.00^a^
F.LDL	66.37 ± 4.06	32.32 ± 2.24	49.12 ± 3.50	0.00^a^

*SD* standard deviation, *F* Fasting, *TG* Triglyceride, *HDL* High Density Lipoprotein. *LDL* Low Density Lipoprotein

^a^Statistically significant

Table 5 shows criteria of metabolic syndrome. There is 3.3% of females with sickle cell disease have triglycerides level ≥ 150 mg/dl.

**Table 5:** Criteria of Metabolic Syndrome among Sickle Cell Disease and control group According to National Cholesterol Education Program-Adult Treatment Panel III

	Proportion at Risk-control*		Proportion at Risk-patients*	
	Male (*n* = 13)	Female (*n* = 17)	Male (n = 13)	Female (n = 17)
Waist circumference (cm)	0.0%	0.0%	0.0%	0.0%
Triglycerides (mg/dl)	0.0%	7.3%	0.0%	3.3%
HDL (mg/dl)	10.3	14.6	93.3%*	100%*
Systolic BP mmHg	0.0%	0.0%	3.3%	3.3%
Diastolic BP mmHg	0.0%	0.0*	3.3%	0.0%
Fasting Glucose (mg/dl)	15.4%	19.3%	6.7%	0.0%
Metabolic Syndrome	0.0%	3.3%	0.0%	3.3%

About 93.3% of males and 100% of females with SCD have HDL level < 40 mg/dl, while 10.3% of males and 14.6% of females HbAA have HDL level < 40 mg/dl.

There is 3.3% of males and 3.3% of females with SCD have systolic blood pressure ≥ 130 mmHg. While 3.3% of males with SCD have diastolic blood pressure ≥ 85 mmHg and 6.7% of males with SCD have fasting blood glucose level ≥ 110 mg/dl.

Only one patient with SCD has a metabolic syndrome (SBP = 160 mmHg, Fasting HDL = 23 mg/dl and Fasting TG = 176 mg/dl). In contrast to one participant from the control group has a metabolic syndrome (FBS = 120 mg/dL, HDL = 32 mg/dL and TG = 150 mg/dL).

## Discussion

Sudan is one of the most populous countries of sickle cell disease. Some literature investigates the risk factors of metabolic syndrome development among this group of patients [2]. In this study the mean waist circumference in SCA patients was not differ among males and females (Data not shown) which is far away lower to be a risk factor for metabolic syndrome to develop: waist circumference must be ≥102 cm (male), ≥ 89 cm (female) [2]. The mean waist circumference was not significantly differing between patients and control groups. A study done by Uche et al., (2017) on 58 patients with SCA found no significant difference of mean waist circumference between patients and control group [4]. Their results was confirmed by study performed by Shawn M Bediako et al. (2015) among 49 adult sickle cell patients [2]. The researchers proposed that nearly half of participants in that study were overweight and had dietary saturated fat intake levels that exceeded both the national average and US Dietary Guidelines [2].

Sickle cell anemia patients complain of hyper metabolic state due to increase circulating pro-inflammatory markers (C- reactive protein and plasma interleukins) and increase resting energy expenditure these two factors decreasing the BMI and waist circumference [8].

The mean hip circumference was significantly lower in SCA compared to control as shown in Table 3. Uche et al., (2017) in their study on 58 SCA patients found significant decrease in hip circumference compared to control which is comparable with our results [4]. This lower hip circumference could be attributed to recurrent episodes of avascular necrosis of the femoral head [4].

The first to describe the relationship between sickle cell and blood pressure were Johnson and his colleagues, in 1981 [9]. In our study the brachial systolic blood pressure in SCA patients was not significantly different from that of the controls (*p* value = 0.4), but the diastolic blood pressure was significantly lower in the patients group compared to controls (p value < 0.05), and the MAP was also found to be significantly lower in patients group compared to control (p value < 0.05).

Several studies conducted among sickle patients showed low blood pressures compared to control group [8–11]. Another study done in neighbour country –Kenya- by Ayuo et al., (1993) reported comparable findings to ours, in which the systolic blood pressure found to be comparable with that of the controls, while the diastolic blood pressure was significantly lower than that of the controls [12]. The mean blood pressure among SCA patients in current study was (106/65 mmHg), which is far away lower to be a risk factor for metabolic syndrome; which is by definition must be ≥130/85 mmHg [3]. In our study 3.3% of males and 3.3% of females with SCA have systolic blood pressure ≥ 130 mmHg, while 3.3% of males have diastolic blood pressure ≥ 85 mmHg and they could be consider as risk groups. Recent studies found that SCA patients may develop low blood pressures due to hypoxia of the chronic anaemic state; the hypoxia causes vasodilatation leading to lower peripheral resistance and reducing systemic blood pressure [10]. In conclusion, our findings support previous studies of relatively lower arterial blood pressure in patients with SCA.

Nowadays some studies investigated the effect of SCA on patient’s lipid profile. In our study, fasting cholesterol level was not significantly higher in patients group compared to control group. Fasting triglycerides was not significantly low in patients group compared to the control group. F.HDL was significantly lower in patients compared to control group.

Padaro et al.,(2016) found significant decrease in cholesterol and HDL levels in Hb SS patients compared to their controls [13, 14]. The researchers also found no significant difference in TG between patients and control groups [15, 16].

Some literatures investigate the derangement of haemoglobin level with erythropoietin production among SCA patients, which is the main factor for cholesterol utilization for red cells membrane formation [14]. In current study, there is insignificant increase in cholesterol level among SCA, which is incomparable with recent studies; we assumed that this difference in our results could be due to derangements of haemoglobin level with erythropoietin production in these patients.

One of the criteria to diagnose metabolic syndrome is dyslipidaemia: TG ≥ 150 mg/dl or HDL < 40 mg/dl (male), < 50 mg/dl (female) [3]. In our study, the mean TG level was 77.3 mg/dl, which is low to be a risk factor for metabolic syndrome. The mean HDL level was 33.3 mg/dl (men), 27.82 mg/dl (women), which it could be consider as a risk factor for metabolic syndrome. Only 6.7% of males have HDL level ≥ 40 mg/dl.

In this study we found that the mean fasting blood glucose level was significantly lower in patients group in comparison to control as shown in Fig. 1.

A study done on 45 sickle cell patients by Yavropoulou,M.P et al.,(2016) found no significant changes in FBG between patients and control groups [17]. Another study done by A.I.Alsultan et al.,(2010) found significant higher FBG in patients compared to control [5]. The researchers assumed that increase fasting blood glucose level occurs as a consequence of insulin resistance develops among SCA patients [5]. Insulin resistance is defined as an organ failure to respond to insulin and consequently increases circulating blood glucose [5]. Sickled haemoglobin is associated with over-production of reactive oxygen species (ROS) [5]. ROS may interfere with insulin signalling at various levels, impairing insulin uptake through a direct effect on insulin receptor function [18]. ROS may reduce glucose uptake by inhibiting the translocation of GLUT4 (glucose transporter 4) to the plasma membrane leading to increase serum blood glucose level [19]. One of the criteria to diagnose metabolic syndrome is fasting plasma glucose ≥110 mg/dl [20].

In this study, only 6.7% of adult male patients have FBG level ≥ 110 mg/dl, Table 5. They may consider as a group of risk of metabolic syndrome.

Secondary gout is a well-recognised complication of increased nucleic acid catabolism in red cells turnover among SCA [21]. In this study we found the mean serum uric acid level was insignificantly differ between Hb SS patients and control group. Only 13.3% of patients have uric acid level greater than 6.8 mg/dl. Previous studies done in Iraq and Brazil found no significant difference in uric acid level between sickle cell patients and their matched control [20, 21].

Sickle cell anaemia nowadays categorized as syndrome rather than a disease. It affects many organs and can be consider as a life threatening condition. These patients may die younger than other population may be due to complications of the disease. This study added new insight to focus on metabolic syndrome development among SCD patients as a complication of the disease. And my shift focus to new therapy strategies to improve patients’ quality of life and reduce both mortality and morbidity.

## Conclusions

There was significant decrease in HDL,DBP, and hip circumference values among SCD patients with no significant difference in cholesterol, triglycerides, SBP, and MAP waist circumference values among SCA patients compared to control.
